# Molecular detection of phenol-soluble modulin-mec (PSM-mec) in Staphylococcus aureus clinical isolates from Federal Medical Center Birnin Kebbi, North-West, Nigeria

**DOI:** 10.3205/dgkh000538

**Published:** 2025-03-06

**Authors:** Isah Musa Maishanu, Adeshina O. Gbonjubola, Hussaini Mujahid, Busayo O. Olayinka

**Affiliations:** 1 Kebbi State University of Science and Technology Aliero, Nigeria; 2 Ahmadu Bello University Zaria, Kaduna, Nigeria; 3 Ummaru Musa Yaradua University Katsina, Nigeria

**Keywords:** Staphylococcus aureus, phenol soluble modulin-mec, panton valentine leucocidin, virulence genes

## Abstract

**Aim:**

This study was carried out to isolate and detect virulence genes associated with *Staphylococcus (S.) aureus* clinical isolates from the Federal Medical Center Birnin Kebbi, Nigeria.

**Methods:**

To obtain *S. aureus* isolates, samples were taken from urine, sputum, blood and wound sources. *S. aureus* was phenotypically identified using Microgen staph ID system and PSM-mec and PVL genes were detected using polymerase chain reaction (PCR).

**Results:**

A total of 48 non-duplicate *S. aureus* isolates were obtained (21 from wound swabs, 7 from blood, 15 from urine, and 5 from sputum). From the 14 *S. aureus* isolates examined by PCR, the most abundant gene was *PSM-mec* (42.8%), while the PVL was the least abundant with 21.4%.

**Conclusion:**

Because it gives highly specific and accurate results, it is essential to use the PCR technique to detect *S. aureus* virulence determinants as well as PSM-mec and PVL as targets for antimicrobial agents.

## Zusammenfassung

**Ziel:**

Diese Studie wurde durchgeführt, um Virulenzgene zu isolieren und zu erkennen, die mit klinischen* S. aureus*-Isolaten des Federal Medical Center Birnin Kebbi assoziiert sind.

**Methode:**

120 *S. aureus*-Isolate wurden aus Urin, Sputum, Blut und Wunden gewonnen. *S. aureus* wurde phänotypisch mithilfe des Microgen Staph ID-Systems identifiziert. Die Gene PSM-mec und PVL wurden mithilfe der Polymerase-Kettenreaktion (PCR) nachgewiesen.

**Ergebnisse:**

Es wurden 48 *S. aureus*-Isolate gewonnen (Wundabstrich 21, Blut 7, Urin 15 und Sputum 5). Von 14 mittels PCR untersuchten *S. aureus* war das am häufigsten vorkommende Gen PSM-mec (42,8%), während das PVL-Gen in 21,4% nachgewiesen wurde.

**Schlussfolgerung:**

Die PCR-Technik muss unbedingt verwendet werden, da sie für die Erkennung von *S. aureus*-Virulenzfaktoren hochspezifische und genaue Ergebnisse liefert und die Erkennung von PSM-mec und PVL neue Targets für antimikrobielle Wirkstoffe darstellen kann.

## Introduction

Being one of the “ESKAPE” organisms, *Staphylococcus (S.) aureus* poses an increasing hazard to human health due to its ability to produce a range of serious nosocomial infections [1]. The relationship among bacterial evolution, host factors, and virulence determinants has been the focus of recent clinical research [2], [3]. The virulence determinants found in *Staphylococcus aureus* comprise staphylokinase, hyaluronidase, lipase, nuclease, hemolysin, leukocidin, and invasive proteases. Leukocidin induces inflammatory reactions by disrupting skin, mucosal cells, and, among the host blood cells, leukocytes [4]. The genes that codes for leukocidin are PSM-mec, luk-F/-S-PV, lukE, lukM, and PSM-α. Due to the presence or lack of mobile genetic elements (MGEs), which are made up of genes encoding for toxins and other virulence factors, the organism’s potential for virulence varies greatly amongst isolates. Antibiotic resistance determinants and virulence factors proliferate as a result of the rich diversification of the naturally adapted mobile genetic element *Staphylococcus* cassette chromosome mec (SCCmec), which is responsible for the stable maintenance of the core genome environment. Unlike several other bacterial pathogens, which frequently depend on just one or a few toxins to cause illness, *S. aureus* generates an incredible variety of virulence factors. These comprise a wide range of protein and non-protein components that facilitate host colonization during infection, as well as an abundance of toxins and immune evasion mechanisms. *Staphylococcal* cassette chromosome mec (*SCCmec*) elements contain PSM-mec, as well as regulatory factors, recombinase genes, mecA, and other resistance genes [5], [6]. The sole known virulence determinant associated with these determinants is the PSM-mec. It has been discovered in *S. aureus* SCCmec types II, III, and VIIIK [7]. Multiple cell surface and secreted virulence factors mediate the pathogenesis of *S. aureus*. One such virulence factor is called Panton-Valentine leukocidin (PVL), an extracellular protein with dermonecrotic and leucocidal properties, which is expressed by the genes luk-S-PV and luk-F-PV. It is cytotoxic to macrophages, monocytes, and neutrophils in mammals. Community-acquired methicillin-susceptible *S. aureus* (CA-MSSA) and community-acquired MRSA (CA-MRSA) strains can both produce the toxin. In addition to causing SSTIs, *PVL*-positive strains have been connected to purpura fulminans, necrotizing pneumonia, bacteremia, and septic arthritis, among other serious diseases [8]. Detecting PSM-mec and PVL genes in *S. aureus* clinical isolates from the Federal Medical Centre Birnin Kebbi was the aim of the current investigation.

## Materials and methods

### Collection and authentication of bacterial isolates 

A total of 120 presumptive staphylococcal isolates were collected from wound swabs, urine, blood and sputum. Following conventional microbiological protocols, the isolates were cultivated and identified using the Microgen Staph ID system (.Microgen bioproducts Ltd, UK). 

### DNA extraction 

Each sample was grown for an entire night at 33°C for 24 hrs on Mueller-Hinton agar plates. After that, 3 ml of sterile lysogeny broth medium were used to placed each single bacterial colony (Oxoid, Hampshire, UK), which was then cultured for eight hours at 33°C with vigorous shaking. Next, DNA was extracted using the Hipure Bacterial DNA Kit (Magen, Guangzhou, China) in compliance with the manufacturer’s instructions.

### Amplification of virulence genes using PCR 

PCR was used to amplify PSM-mec and PVL, as previously reported by Jiang [9] in 14 isolates of *S. aureus*. Table 1 (Tab. 1) lists the target genes, primer sequences, and target segment of the PCR products. The PCR techniques were performed in a final volume of 25 µL of reaction mixture that contained 50 ng of genomic DNA, 20 pmol of each primer, and 12.5 µL of 2×Taq PCR Master Mix (Tiangen Biotech, China: 0.1 U of Taq polymerase/µL, 0.5 mM dNTP each, 20 mM Tris-HCl/pH 8.3, 100 mM KCl, 3 mM MgCl_2_). The denaturation process was completed in 3 minutes at 94°C, followed by 30 cycles of 30 seconds each at 94°C for denaturation, 30 seconds of annealing at 55°C 1 minute of primer extension at 72°C, and 5 minutes of final extension at 72°C.

### Statistical analysis 

The statistical software for social sciences (SPSS) version 21 was used to analyze the data. The analysis employed descriptive statistics such as percentages.

## Results

Forty-eight (48)* S. aureus* isolates were obtained during the course of a 6-month study period. The majority of *S. aureus* isolates were from wound (43.7%), urine (31.25%) and blood (12.5%) while sputum was (10.4%). A breakdown of the prevalence of (17.5%) in wound samples was recorded as shown in (Table 2 (Tab. 2)). 

**Figure 1:**
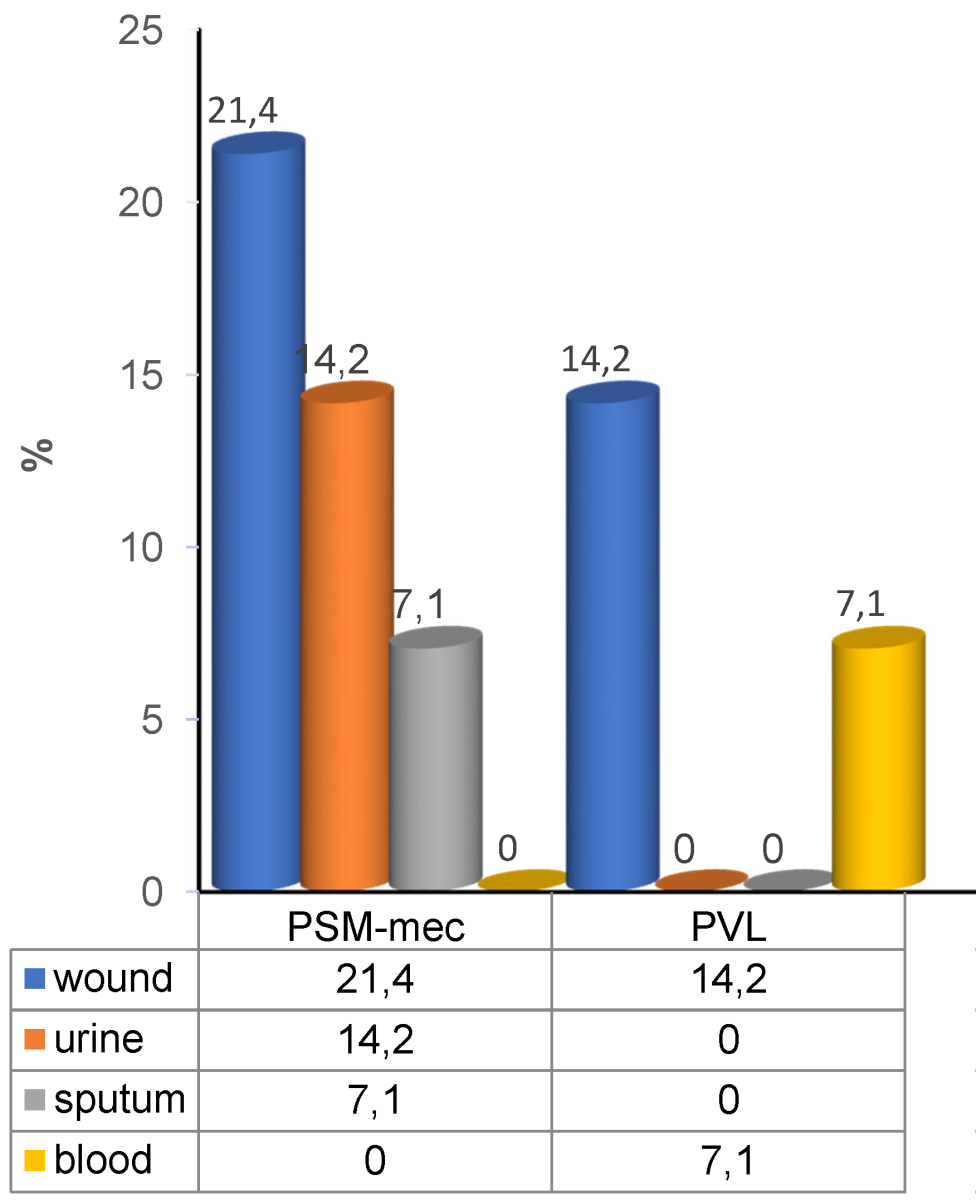
Percentage of virulence genes in *S aureus* from various clinical specimens. (PVL=panton valentine leucocidin, PSM-mec=phenol soluble modulin-mec)

The prevalence of PVL and PSM-mec genes in 14 of the *S. aureus* isolates was 21.4% (3/14) and 42.8% (6/14), respectively. The highest proportion of PSM-mec genes was detected in the *S. aureus* isolates from wound swabs, followed by urine, while PVL genes were also detected in mostly in wound swabs, followed by blood (Figure 1 (Fig. 1)).

## Discussion

In the community, hospital, and environmental settings, virulence factors are an essential component of pathogenic invasion that results in staphylococcal infection. Global reports indicate that *S. aureus* possesses a rich diversity of virulence-associated genes [10], [11]. The virulence analysis was focused on detecting PVL and PSM-mec genes. Research has shown that the prevalence of PVL in *S. aureus* isolates derived from clinical specimens varies greatly between nations, with prevalences as high as 57% observed in isolates from west African countries and 9.7% in England [12], [13]. We investigated the prevalence of PVL genes in 14 clinical samples that tested positive for *S. aureus*. A prevalence of 33.0% was detected out of the 14 *S. aureus* isolates that underwent investigation for the PVL gene. This is substantially greater than what was found in some studies conducted in Nigeria, with 10.7% [14], 11.2% [15], [16] and 13% [17], but was similar to others, e. g, in Jos Nigeria with 31.3% [18], 34% in Southwest Nigeria [19] and 39.35% at Obafemi Awolowo University [20]. In contrast, our results were lower than in Maiduguri, Nigeria, which had 52.1% [21], Gambiya 61% [22], Sudan 58% [23], Iran 56% [24] and India 61% [25]. 

The virulence factor PSM-mec belongs to the class of amphipathic, alpha-helical peptide poisons known as phenol-soluble modulins (PSM). All known PSMs are core-genome encoded, with the exception of the PSM-mec, which is present in specific subtypes of SCCmec methicillin-resistant mobile genetic elements, discovered in methicillin-resistant *S. aureus*. According to Wang et al. [25], the alpha-type phenol-soluble modulins (PSMs), which are novel cytolytic peptides, are encoded in an operon present in every strain of *S. aureus* that has been sequenced. Recent studies suggest that PSM synthesis is inhibited by the transcription and translation products of PSM-mec, which are present in the HA-MRSA mobile genetic elements SCCmec-II and -III [26]. Queck et al. [27] discovered that PSM-mec had a positive impact on the pathogenicity of the HA-MRSA strain MSA890. Also, the translational product of PSM-mec was found to be more prevalent than that of other PSMs. In the present study, only 42.8% of the 14 MRSA isolates that underwent PCR analysis carried the PSM-mec gene. The majority of HA-MRSA carries the PSM-mec gene, which is present in type-II and type-III SCCmec and regulates *S. aureus* pathogenicity [28]. The* S. aureus* PSM-mec gene, which is necessary for MRSA colonization and pathogenesis, is still not well understood. Given the essential roles of the PVL and PSM-mec genes, these virulence factors may be potential candidates for consideration in vaccines that combat MRSA strains. 

## Conclusions

*S. aureus* isolated from clinical samples in Nigeria possessed the PSM-mec and PVL genes. An understanding of the links between virulence and resistance would help to lessen the impact of *S. aureus* infections, given the high prevalence of infections produced by this pathogen and its significance in human medicine. To obtain deeper insight into the various virulence and resistance mechanisms employed by this pathogen and their interactions, additional research is required.

## Notes

### Competing interests

The authors declare that they have no competing interests.

### Ethical approval 

Ethical clearance was obtained from the ethical committee of Federal Medical Center Birnin Kebbi, Nigeria to enable collection of Staphylococcal clinical isolates from medical microbiology unit of the hospital.

### Funding

None. 

### Acknowledgments

We are very grateful to members of staff of Microbiology unit, Federal Medical Center Birnin Kebbi and that of Central Research Laboratory, Department of Veterinary Microbiology Usman Dan Fodio University Sokoto for their cooperation and assistance during the study.

## Figures and Tables

**Table 1:**
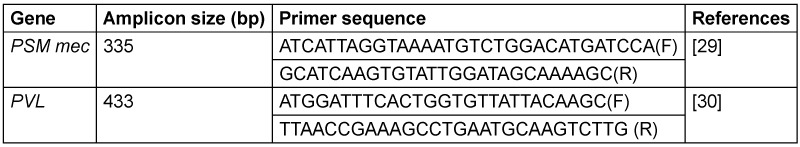
The primers used and their nucleotide sequences

**Table 2:**
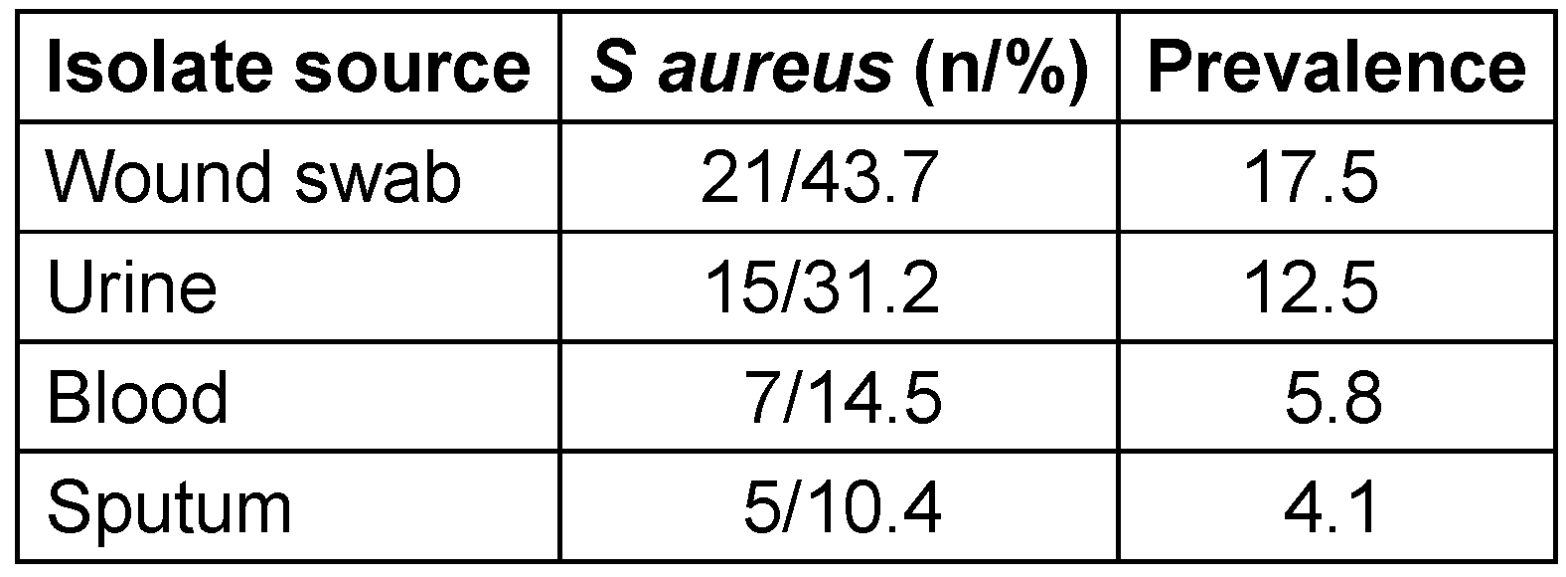
Prevalence of *Staphylococcus aureus* isolates by specimen
